# Evaluation of various kinetic parameters of CA-125 in patients with advanced-stage ovarian cancer undergoing neoadjuvant chemotherapy

**DOI:** 10.1371/journal.pone.0203366

**Published:** 2018-09-06

**Authors:** Yong Jae Lee, In Ha Lee, Yun-Ji Kim, Young Shin Chung, Jung-Yun Lee, Eun Ji Nam, Sunghoon Kim, Sang Wun Kim, Young Tae Kim

**Affiliations:** Department of Obstetrics and Gynecology, Institute of Women’s Medical Life Science, Yonsei University College of Medicine, Seoul, Korea; University of Nebraska Medical Center, UNITED STATES

## Abstract

Various kinetic parameters of serum CA-125 have been reported to have better correlation with outcomes for patients treated with neoadjuvant chemotherapy (NAC). This study aimed to compare the available kinetic parameters of serum CA-125 in an external cohort of advanced-stage ovarian cancer. Using the cancer registry databases from the Yonsei Cancer Hospital, we retrospectively reviewed 210 patients with advanced-stage ovarian cancer, treated with NAC followed by interval debulking surgery. We compared area under the receiver-operating characteristics curves (AUCs), false negative rate, and negative predictive value (NPV) using 10 different models for optimal cytoreduction and platinum resistance. In addition, we compared incremental AUC for progression-free survival (PFS) and overall survival (OS). No gross residual tumor was observed in 37.0% and residual tumors <1 cm in 82.2% of patients. No model using CA-125 kinetic parameters had an AUC higher than >0.6 for predicting optimal cytoreduction. After adjusting for age, BMI, disease stage, and histologic subtypes, all models had an AUC >0.70 for predicting platinum resistance. However, no model had a high enough NPV (highest value = 82.0%) to avoid chemotherapy futility. For survival outcomes, no model had an incremental AUC >0.70 for predicting either PFS or OS. None of the proposed serum CA-125 kinetic parameters showed high accuracy in predicting optimal cytoreduction, platinum resistance, or survival in patients receiving NAC. For advanced-stage ovarian cancer treated with NAC, there is a need to discover reliable biomarkers to better stratify patient response groups for optimal treatment decision-making.

## Introduction

Epithelial ovarian cancer is the leading cause of death among all gynecological malignancies in Korea [1, 2]. The standard therapy of advanced-stage ovarian cancer includes primary debulking surgery (PDS), followed by platinum-based combination chemotherapy. Recently, several phase 3 clinical trials showed that neoadjuvant chemotherapy (NAC) followed by interval debulking surgery (IDS), was not an inferior treatment course compared to PDS for treating advanced-stage ovarian cancer [3–6]. NAC followed by IDS has gained popularity as an alternative approach that may reduce perioperative morbidity.

A potential advantage of the NAC approach is that it can provide the treating clinician with early information regarding chemotherapy response using clinical, radiological, and tumor markers. As chemo-sensitivity during NAC has been a well-known prognostic factor for survival in advanced-stage ovarian cancer [7, 8], identifying a patient as either a good responder or poor responder to NAC before IDS is important to triage further adjuvant treatment.

Cancer antigen-125 (CA-125) is the only serological biomarker in routine use for evaluating disease status and monitoring the efficacy of chemotherapy for the management of patients with epithelial ovarian cancer. In the neoadjuvant setting, where the effect of surgery is excluded, the value of CA-125 is expected to be correlated with NAC response. Therefore, 10 different models for the evaluation of CA-125 regression during NAC, as a predictor of optimal cytoreduction during IDS or a prognostic factor for survival, have been proposed in the literature [9–18]. Various kinetic parameters of CA-125 have been evaluated for optimal cytoreduction, platinum resistance, and patient survival.

Identifying a patient as either a good or poor responder to treatment can be useful at two levels. First, such a prediction for optimal cytoreduction at IDS may offer better patient counseling by preventing futile surgery. Second, postoperative adjuvant chemotherapy (POAC) could be tailored according to the prediction of platinum resistance. For poor responders, clinical trials with other biologic agents could be applied with molecular profiling instead of conventional chemotherapy.

The aim of this study was to compare 10 previous studies using various kinetic parameters of serum CA-125 as a predictive or prognostic factor, as well as to validate the results in an external cohort of advanced-stage ovarian cancer treated with NAC.

## Materials and methods

### Study population

The electronic medical records of patients diagnosed between 2008 and 2016 with epithelial ovarian, fallopian tube, and primary peritoneal carcinoma, who received NAC followed by IDS at the Department of Obstetrics and Gynecology, Severance Hospital, Yonsei University College of Medicine, were retrospectively analyzed. The disease stage of these patients was based on biopsy or cytology and the extent of disease according to radiological imaging. The present study was reviewed and approved by the institutional review board at Severance Hospital, Yonsei University Health System, Seoul, Korea.

In brief, the inclusion criteria were as follows: (1) histopathologically confirmed FIGO stage III or IV ovarian, fallopian tube, and primary peritoneal carcinoma; (2) patients who underwent IDS following NAC; (3) patients having received more than 1 cycle of NAC before IDS. The exclusion criteria were as follows: patients with FIGO stage I or II (N = 5), patients who have not received IDS after NAC (N = 2), those who were lost to follow-up (N = 2), and patients who have not received platinum-based chemotherapy (N = 4). Ultimately, the final study population comprised 197 female patients (S1 Fig).

The characteristics of the study patients are shown in Table 1. We retrospectively collected data from medical records, including age, body mass index (BMI), disease characteristics (histology, FIGO stage), residual disease after IDS, date of progression or recurrence, date of last follow-up, and patient disease status at last contact. All patients underwent surgery with intent to achieve complete cytoreduction (no gross residual disease). Surgery was considered “radical” if the procedures included upper abdomen surgery, bowel resection, or video-assisted thoracoscopic surgery (VATS). Diagnosis of recurrence was based on radiological imaging, and/or histology diagnosis ultimately determined by gynecology oncologists. The treatment-free interval (TFI) was defined as the interval from the end of platinum-based chemotherapy to the first recurrence. A threshold value for TFI of 6 months was applied to qualify a patient as having platinum-sensitive disease.

**Table 1:** Patient characteristics (N = 197).

Characteristics	
Median age, years (range)	57 (27–80)
Median CA-125 level, U/mL (range)	1825.7 (44.3–30000.0)
Median CA-125 level before IDS, U/mL (range)	45.6 (4.0–7913.1)
FIGO stage, n (%)	
IIIB	7 (3.6%)
IIIC	45 (22.8%)
IVA	89 (45.2%)
IVB	56 (28.4%)
Histologic type, n (%)	
HGSC	180 (91.4%)
Non-HGSC	17 (8.6%)
Radical surgery^a^, n(%)	
None	
Any radical surgery	
Residual disease after IDS, n (%)	
No gross	72 (36.6%)
≤0.5cm	63 (32.0%)
0.5-1cm	27 (13.7%)
1-2cm	5 (2.5%)
>2cm	8 (4.1%)
NA	22 (11.2%)
Cycles of total chemotherapy, n (%)	
<6	8 (4.1%)
≥6	189 (95.9%)

^a^Radical surgery includes any of following: bowel surgery, video-assisted thoracoscopic surgery, splenectomy, liver resection, supraclavicular fossa resection, ureter resection, and others.

BMI, body mass index; CA-125, cancer antigen 125; FIGO, International Federation of Gynecology and Obstetrics; HGSC, high-grade serous carcinoma.

### Ethical considerations

This retrospective study was approved by the Institutional Review Board of Severance Hospital at Yonsei University College of Medicine (IRB No. 4-2018-0518). The patient records were anonymized and de-identified prior to analysis.

### Measurement of CA-125

For each patient, CA-125 levels from pre-NAC to post-POAC were recorded. CA-125 levels were measured initially (before the first cycle of NAC), then every 3 weeks before chemotherapy. The B-R-A-H-M-S CA-125 II KryptorR technique, an automatic immunofluorescence analysis kit used to measure CA-125 in the serum or plasma, was used to assay CA-125. A concentration of CA-125 ≤35 UI/mL was considered normal.

### Statistical analysis

Discrimination was quantified using the area under the curve (AUC) from receiver operating characteristic (ROC) analysis. The AUC corresponds to the overall predictive validity. The AUC reflects the ability of a test to discriminate between a diseased and a non-diseased subject across all possible levels of positivity. False negative (FN) rates and negative predictive values (NPVs) were also evaluated. We compared C-statistics for survival. The expected value of the C-index is 0.5 for a model with no explanatory power. C-index achieves its maximum value of 1.0 when the model assigns a lower probability of occurrence to all cases with no adverse events than to any cases with an adverse event. Progression-free survival (PFS) was defined as the interval between the date of diagnosis and the date of first recurrence. Overall survival (OS) was defined as the interval between the date of diagnosis and the date of death. Statistical analyses were conducted with SAS software (version 9.2; SAS Institute, Cary, NC). For all analyses, P < 0.05 was considered statistically significant.

## Results

### Description of various kinetic parameters of CA-125

Ten models developed to predict optimal cytoreduction, platinum resistance, and survival were identified from the medical literature using PubMed. Seven models were designed to predict optimal cytoreduction and 2 models were derived to predict platinum-resistance. All models evaluated PFS and OS. Nine models classified patients as being either good or poor responders for NAC (binary models). The most important characteristics of each model are summarized in Table 2.

**Table 2:** Description of regression models to predict CA-125 values during neoadjuvant chemotherapy.

No.	Models	Stage	NAC cycles, median	N	Chemotherapy regimen	CA125 before NAC (UI/mL)	CA125 cut-off (UI/mL)	Outcomes
1	Zeng et al.(2016)	IIIC-IV	1–3 (97.5%) (range, 1–7)	118	Paclitaxel + Platinum	1814 (56.6–56541.0)	Before IDS ≤200	Complete cytoreduction Chemotherapeutic sensitivity PFS, OS
2	Pelissier et al.(2016)	IIIC-IV	5.6 (range, 4–6)	142	Platinum-based	2128.7 (15–16275)	After the 3rd cycle of NAC <35	Platinum sensitivity PFS, OS
3	Morimoto et al.(2016)	III-IV	4 (range, 3–6)	139	Paclitaxel+carboplatin	1578 (33.1–26606)	Before IDS ≤25.8	Complete cytoreduction PFS, OS
4	Mahdi et al.(2015)	IIIC-IV	4 (range, 3–6)	115	Platinum-based	905.5 (47–20,000)	Reduction ≥90% (Before NAC/ Before IDS)	Complete cytoreduction PFS, OS
5	Pelissier et al.(2014)	IIIC-IV	5.6 (range, 1–9)	148	Taxane + platinum	2122 (15–26,220)	After the 3rd cycle of NAC <75	Complete cytoreduction PFS, OS
6	Furukawa et al.(2013)	III	3	75	Taxane + platinum	Complete IDS: 639 (57–6,593) Non-complete IDS: 1427 (45–10,989)	Before IDS ≤20	Complete cytoreduction PFS, OS
7	Rodriguez et al.(2012)	IIIC-IV	3 (range, 1–8)	103	Taxane + platinum	1749 (38–10,150)	Before IDS ≤100	Complete cytoreduction PFS, OS
8	Vasudev et al.(2011)	III-IV	Not reported (range, 3–6)	63	Platinum-based	1250 (53–16,398)	Half-life <12 days, >18 days Regression coefficient ≥-0.039	Optimal cytoreduction PFS, OS
9	Le et al.(2008)	II-IV	Not reported	90	Taxane + platinum	Not reported	Before IDS <35	PFS, OS
10	Tate et al.(2005)	IIIC-IV	4.5	50	Platinum-based	Good responder: 2843 Poor responder: 3697	Regression coefficient >-0.039	3-year survival PFS, OS

NAC, neoadjuvant chemotherapy; IDS, interval debulking surgery; PFS, progression-free survival; OS, overall survival.

Various kinetic parameters of CA-125 were obtained from published formulas. Before applying the model to the patient data to calculate individual kinetic parameters of CA-125, we investigated whether a patient’s characteristics made them eligible for the specific model. Patients were only evaluated in each prediction model based on the inclusion criteria for the specific prediction model. Most models were designed to calculate CA-125 for FIGO stage III or IV [9–13, 16, 18]. Model 6 was designed to calculate CA-125 in patients with FIGO stage III [14], and model 9 was designed to calculate CA-125 in patients with FIGO stage II–IV disease [17]. In contrast, most models were designed to calculate CA-125, regardless of the histology of the disease [9–17]. Model 10 was designed to calculate CA-125, excluding clear cell carcinoma and mucinous adenocarcinoma [18]. All models reported cut-off values that allowed for the identification of patients who were good responder to chemotherapy. The cut-off values for each model are shown in Table 2. In model 1, 3, 6, and 7, the CA-125 value before IDS was used [9, 11, 14, 15], and model 2, 5, 9 used the CA-125 value after 3 cycles of NAC [10, 13, 17]. Model 8 and 10 used regression coefficients, which were calculated using CA-125 levels from the time of NAC initiation until CA-125 normalization, or the day of IDS [16, 18]. Model 4 used the percent reduction in CA-125 (from pre-NAC to pre-IDS value) [12].

### Prediction for optimal cytoreduction

Among the 197 study patients, 72 (36.6%) had no gross residual disease after IDS. In addition, 162 patients (82.2%) achieved optimal cytoreduction with residual tumors <1 cm after IDS.

All CA-125 models were applied for the prediction of no gross residual tumor and optimal cytoreduction. The number of patients, AUC, FN rate, NPV, and patients classified as good responders are shown in Table 3. None of models using CA-125 kinetic parameters predicted surgical outcome accurately, and no model had an AUC higher than 0.60.

**Table 3:** Evaluation of models to predict residual disease.

Predicting R0 after IDS								
No.	Model	No. of patients	No residual tumor	AUC	95% CI	FN rate	NPV	Patients assigned to the good responder
1	Zeng et al.(2016)	193	68/193 (35.2%)	0.50	0.44–0.56	80.0%	35.1%	39/193 (20.2%)
2	Pelissier et al.(2016)	184	65/184 (35.3%)	0.55	0.48–0.63	34.5%	41.4%	114/193 (59.1%)
3	Morimoto et al.(2016)	193	68/193 (35.2%)	0.57	0.50–0.64	32.0%	43.7%	122/193 (63.2%)
4	Mahdi et al.(2015)	201	72/201 (35.8%)	0.52	0.46–0.58	72.9%	36.9%	52/201 (25.9%)
5	Pelissier et al.(2014)	184	65/184 (35.3%)	0.53	0.46–0.60	58.8%	37.5%	72/184 (39.1%)
6	Furukawa et al.(2013)	193	68/193 (35.2%)	0.56	0.49–0.63	27.2%	43.3%	133/193 (68.9%)
7	Rodriguez et al.(2012)	193	68/193 (35.2%)	0.50	0.43–0.57	68.8%	35.3%	60/193 (31.1%)
8	Vasudev et al.(2011)	194	69/194 (35.6%)	0.51	0.43–0.58	58.4%	36.0%	80/193 (41.5%)
9	Le et al.(2008)	193	68/193 (35.2%)	0.57	0.50–0.64	39.2%	42.4%	108/193 (56.0%)
10	Tate et al.(2005)	194	69/194 (35.6%)	0.50	0.43–0.58	59.2%	35.1%	80/194 (41.2%)
Predicting R1 after IDS								
No.	Model	No. of patients	Residual tumor <1cm	AUC	95% CI	FN rate	NPV	Patients assigned to the good responder
1	Zeng et al.(2016)	193	160/193 (82.9%)	0.51	0.44–0.59	81.8%	82.5%	39/193 (20.2%)
2	Pelissier et al.(2016)	184	155/184 (84.2%)	0.54	0.44–0.64	44.8%	81.4%	114/193 (59.1%)
3	Morimoto et al.(2016)	193	160/193 (82.9%)	0.52	0.42–0.61	39.4%	81.7%	122/193 (63.2%)
4	Mahdi et al.(2015)	201	166/201 (82.6%)	0.55	0.47–0.64	65.7%	84.6%	52/201 (25.9%)
5	Pelissier et al.(2014)	184	155/184 (84.2%)	0.51	0.41–0.61	62.1%	83.9%	72/184 (39.1%)
6	Furukawa et al.(2013)	193	160/193 (82.9%)	0.54	0.46–0.62	56.3%	86.7%	133/193 (68.9%)
7	Rodriguez et al.(2012)	193	160/193 (82.9%)	0.54	0.46–0.62	75.8%	81.2%	60/193 (31.1%)
8	Vasudev et al.(2011)	194	162/194 (83.5%)	0.52	0.42–0.61	56.3%	84.2%	80/193 (41.5%)
9	Le et al.(2008)	193	160/193 (82.9%)	0.51	0.41–0.60	45.5%	82.4%	108/193 (56.0%)
10	Tate et al.(2005)	194	162/194 (83.5%)	0.50	0.41–0.60	59.4%	83.3%	80/194 (41.2%)

### Prediction for platinum-resistance

**Fig 1:**
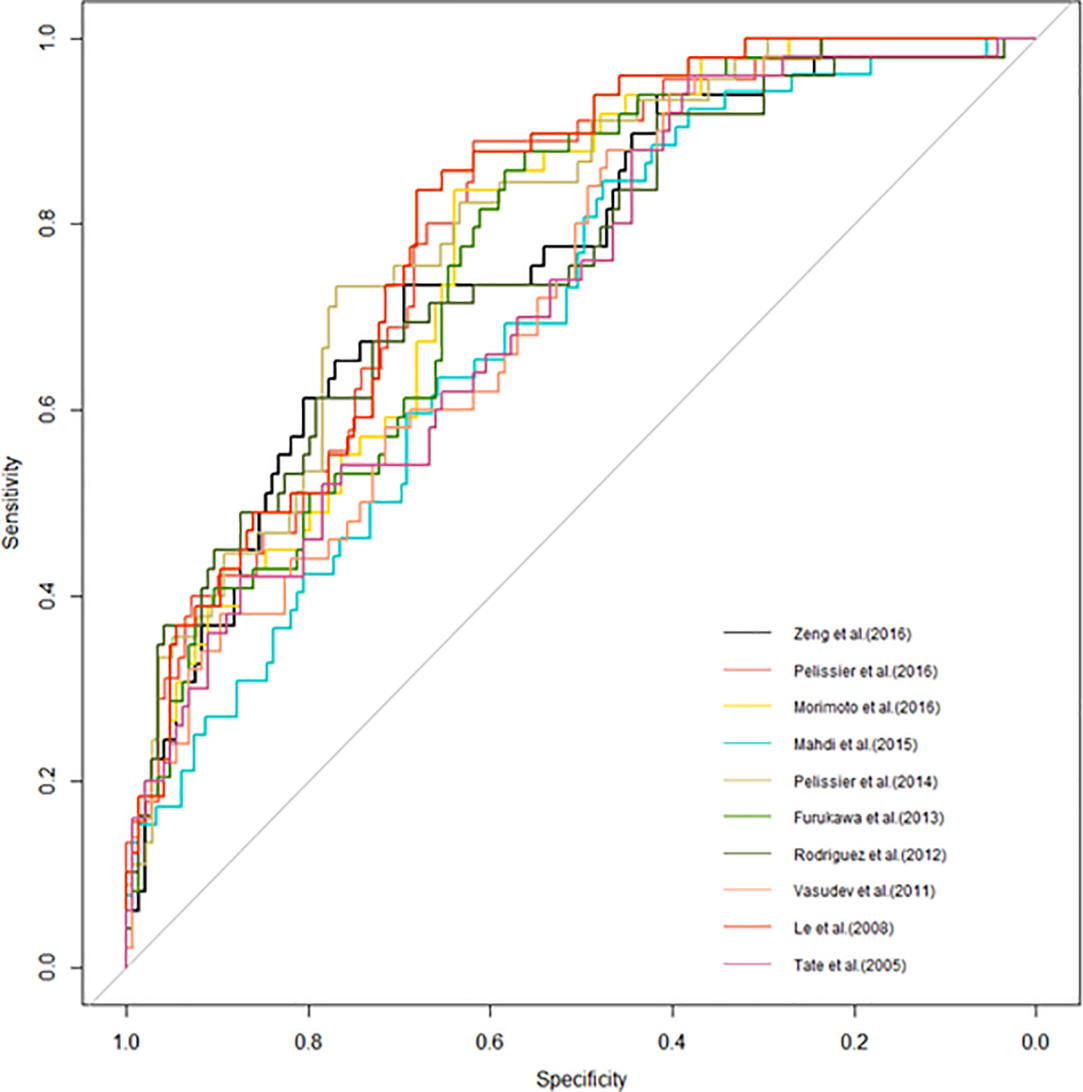
Receiver-operating characteristics curves of 10 models to predict platinum-resistance.

The ROC curves are plotted in Fig 1. When adjusted for age, BMI, disease stage, and histologic subtype, the model with the highest AUC was that reported by Le et al. using CA-125 normalization after NAC, with an AUC of 0.80 (95% confidence interval (CI): 0.734–0.864). The other model gave a performance with AUC values ranging from 0.70–0.79 (S1 Table). However, none of the models had acceptable FN rate (<5%) or enough NPV (>90%).

### Prediction for survival

When adjusting for age, BMI, disease stage, and histologic subtype, all models had a significant prognostic factor for PFS and OS. All models gave performances ranging from 0.65–0.70 for iAUC and the Harrell’s C-index ranged from 0.70–0.76 for PFS and OS (S2 Table). However, none of the 10 models investigated had an iAUC >0.7 or a C-index >0.8 for the prediction of either PFS or OS.

## Discussion

Our study evaluated and compared the performance of 10 models currently available to predict optimal cytoreduction, platinum-resistance, and survival outcomes in patients undergoing IDS after NAC for advanced-stage ovarian cancer. The 10 models were all applied to an independent cohort of advanced-stage ovarian cancer. This study shows the limited usefulness of using CA-125 kinetic parameters for predicting patient outcomes. In particular, none of the 10 models had high accuracy in predicting optimal cytoreduction, platinum-resistance, or survival outcomes.

Ovarian cancer is one of the most chemo-sensitive of all solid tumors, with over 80% of patients showing a response to standard platinum-based chemotherapy. Even if preoperative imaging shows massive ascites and diffuse dissemination, some patients still achieved a nearly complete response with IDS after NAC treatment. In addition, these subgroups had better survival than the poor responder group after NAC. Therefore, identifying good and poor responders after NAC, but before IDS, is an important issue for the following reasons. First, prediction for optimal cytoreduction at IDS may offer patient counseling by preventing futile surgery. Second, POAC could be tailored according to the prediction of platinum resistance. Third, response to NAC could be used as a surrogate for survival. However, there is no reliable biomarker at present other than CA-125 for identifying good responders/poor responders after NAC.

CA-125 is still the only tumor marker that is recommended as a diagnostic or prognostic indicator, as well as for monitoring the efficacy of chemotherapy and disease recurrence after surgery. CA-125 is routinely used to assess response to chemotherapy and, together with cross-sectional radiological findings, is currently used to determine the resectability for IDS in patients with advanced-stage ovarian cancer [19]. Reduction of CA-125 could be used in the neoadjuvant setting to predict chemotherapy response. Patients who show no CA-125 response to NAC often do not undergo IDS and have a poor prognosis [20].

The previous 10 models [9–18] investigated various cut-off values of CA-125 to find good and poor responders among patients who underwent NAC in advanced-stage ovarian cancer. In the models for predicting optimal cytoreduction, it is important to increase the optimization of IDS for good responders, as well as to reduce futile surgery for the poor responders. Therefore, futile surgery can be reduced if the model has a high NPV (>90%), with an FN rate of <5%. Continuing second-line chemotherapy alone can provide reasonable disease control in patients who were unsuitable for optimal IDS in the poor responder subgroup [21]. All models showed poor performance in terms of discrimination in predicting optimal cytoreduction after NAC.

It is important to reduce the incidence of platinum-resistance in the good responder patient subgroup treated with NAC. In the models for predicting platinum-resistance, a low NPV increases the possibility of administering futile chemotherapy. Patients with poor response to platinum-based NAC show a short-term therapeutic effect from chemotherapy and a high probability of platinum-resistant disease. Therefore, clinical trials using other targeted agents with molecular profiling should be considered over conventional chemotherapy. Although CA-125-based parameters showed some accuracy in predicting platinum-resistance in the 10 models, no single model showed a sufficient NPV (highest value = 82.0%).

The 10 models showed moderate accuracy for predicting PFS and OS. The iAUC for predicting PFS ranged from 0.65–0.68, and OS ranged from 0.65–0.70. The iAUC is a weighted average of the AUC over the entire follow-up period, as well as a measure of predictive accuracy of the model during follow-up. None of the 10 models displayed a sufficient iAUC (highest value = 0.70). Harrell’s C-index estimates the ability of the serum CA-125 kinetic parameters to discriminate patients with respect to their recurrence, mortality, and survival. All 10 models showed a moderate Harrell’s C-index for predicting PFS and OS; Harrell’s C-index for predicting PFS ranged from 0.65–0.69 and OS ranged from 0.70–0.76. All models predicting OS showed a relatively high value of Harrell's C-index compared to models used for predicting PFS. These results suggest that survival outcomes are better in the good responder subgroup. In the good responder patient subgroup, NAC may improve overall patient quality-of-life and functional status [22], in addition to provide favorable perioperative morbidity. NAC can result in adequate tumor shrinkage and effectively increase the feasibility of optimal cytoreductive surgery [23]. Furthermore, survival outcomes can be improved by performing the same regimen for POAC, in which efficacy has been shown for NAC. In the poor responder subgroup, even when optimal cytoreduction is achieved and postoperative adjuvant chemotherapy is performed, there is still a high risk of recurrence.

Our study had some limitations. First, the entire patient cohort was not used to assess each model. This was due to not all the models being designed for the evaluation of the response to NAC for the same patients. Most models were designed to evaluate patients with FIGO stage IIIC and IV [9, 10, 12, 13, 15, 18]. Moreover, model 2 and 8 were designed to evaluate patients with FIGO stage III and IV [11, 16], whereas model 9 was designed to predict the response to NAC with FIGO stage II to IV [17], and model 6 was designed to predict the response to NAC in only FIGO stage III [14]. Second, CA-125 was the only variable used, with the cut-off value being set to predict patients that would be good responders in all models.

To the best of our knowledge, this is the first study comparing 10 previous studies using various kinetic parameters of serum CA-125 as a predictive factor in patients of advanced-stage ovarian cancer treated with NAC. None of the proposed kinetic parameters utilizing serum CA-125 showed high accuracy in predicting optimal cytoreduction or platinum-resistance in patients undergoing IDS after NAC. All models showed moderate accuracy for the prediction of survival outcomes. Future prospective studies are required to explore reliable strategies that will allow for the stratification of NAC treatment response groups for predicting optimal cytoreduction at IDS to prevent futile surgery, platinum-resistance to tailor appropriate POAC, and survival outcomes.

## Supporting information

S1 Fig Flow diagram of the study population. NAC, neoadjuvant chemotherapy; FIGO, FIGO, International Federation of Gynecology and Obstetrics.
(TIFF)

S1 Table Evaluation of models to predict platinum resistance. (DOCX)

S2 Table Evaluation of models to predict survival outcomes. (DOCX)
