# Supplementary figures and images for: Evaluation of various kinetic parameters of CA-125 in patients with advanced-stage ovarian cancer undergoing neoadjuvant chemotherapy
Source: PLoS One. 2018 Sep 6;13(9):e0203366. doi: 10.1371/journal.pone.0203366 (PMC6126869; doi:10.1371/journal.pone.0203366)

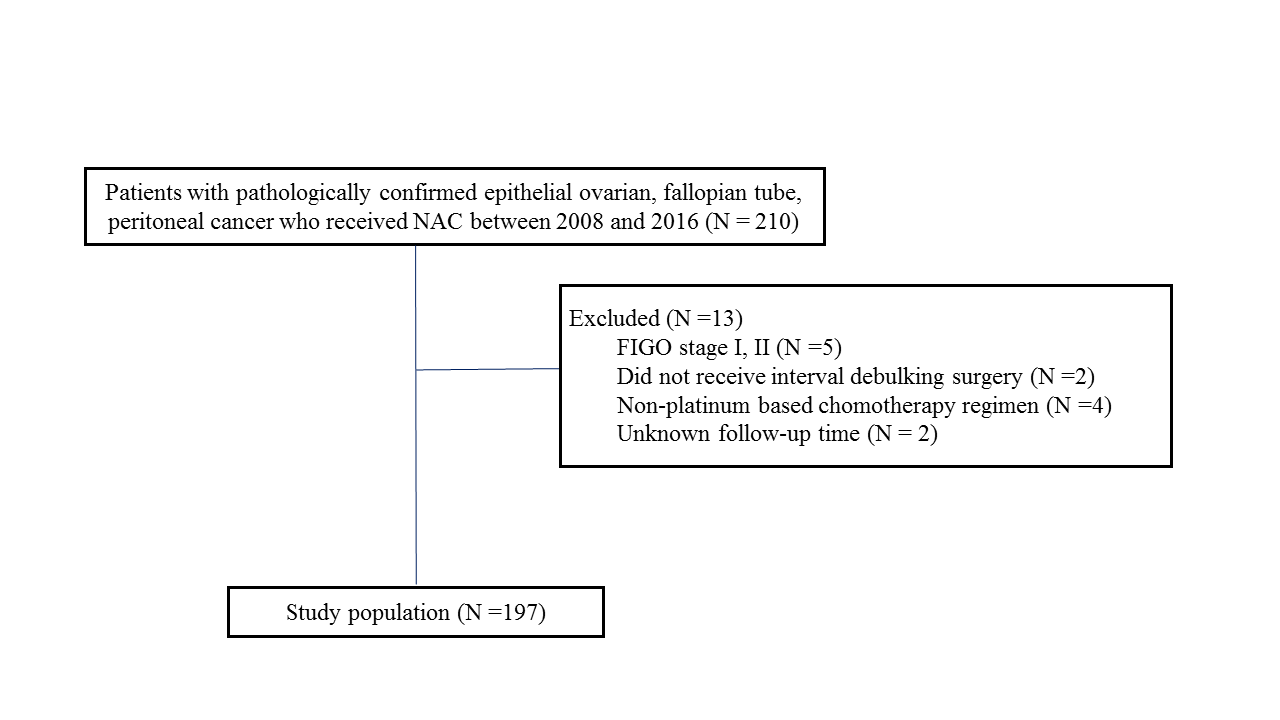

Supplement: S1 Fig — NAC, neoadjuvant chemotherapy; FIGO, FIGO, International Federation of Gynecology and Obstetrics. (TIFF) [file pone.0203366.s001.tiff]
