# Supplementary material for: Evaluation of various kinetic parameters of CA-125 in patients with advanced-stage ovarian cancer undergoing neoadjuvant chemotherapy
Source: PLoS One. 2018 Sep 6;13(9):e0203366. doi: 10.1371/journal.pone.0203366 (PMC6126869; doi:10.1371/journal.pone.0203366)
Supplement: S1 Table — (DOCX) [file pone.0203366.s002.docx]

| No. | Model | AUC | 95% CI | FN rate | NPV |
| --- | --- | --- | --- | --- | --- |
| 1 | Zeng et al.(2016) | 0.76 | 0.68 - 0.84 | 75.51% | 78.61% |
| 2 | Pelissier et al.(2016) | 0.79 | 0.72 - 0.86 | 71.11% | 80.72% |
| 3 | Morimoto et al.(2016) | 0.77 | 0.70 - 0.84 | 73.47% | 79.07% |
| 4 | Mahdi et al.(2015) | 0.70 | 0.62 - 0.78 | 82.69% | 77.01% |
| 5 | Pelissier et al.(2014) | 0.79 | 0.72 - 0.86 | 64.44% | 81.99% |
| 6 | Furukawa et al.(2013) | 0.76 | 0.69 - 0.84 | 71.43% | 79.53% |
| 7 | Rodriguez et al.(2012) | 0.75 | 0.67 - 0.83 | 63.27% | 81.55% |
| 8 | Vasudev et al.(2011) | 0.72 | 0.64 - 0.80 | 80.00% | 77.53% |
| 9 | Le et al.(2008) | 0.80 | 0.73 - 0.86 | 65.31% | 80.95% |
| 10 | Tate et al.(2005) | 0.72 | 0.64 - 0.80 | 78.00% | 77.97% |
