# Supplementary material for: Evaluation of various kinetic parameters of CA-125 in patients with advanced-stage ovarian cancer undergoing neoadjuvant chemotherapy
Source: PLoS One. 2018 Sep 6;13(9):e0203366. doi: 10.1371/journal.pone.0203366 (PMC6126869; doi:10.1371/journal.pone.0203366)
Supplement: S2 Table — (DOCX) [file pone.0203366.s003.docx]

| PFS | | | | | |
| --- | --- | --- | --- | --- | --- |
| No. | Model | iAUC | 95% CI | Harrell's C | 95% CI |
| 1 | Zeng et al.(2016) | 0.65 | 0.61 - 0.70 | 0.66 | 0.61 - 0.71 |
| 2 | Pelissier et al.(2016) | 0.66 | 0.61 - 0.71 | 0.68 | 0.63 - 0.73 |
| 3 | Morimoto et al.(2016) | 0.67 | 0.63 - 0.71 | 0.67 | 0.63 - 0.72 |
| 4 | Mahdi et al.(2015) | 0.65 | 0.60 - 0.69 | 0.65 | 0.60 - 0.69 |
| 5 | Pelissier et al.(2014) | 0.66 | 0.60 - 0.71 | 0.68 | 0.63 - 0.73 |
| 6 | Furukawa et al.(2013) | 0.66 | 0.62 - 0.70 | 0.67 | 0.62 - 0.71 |
| 7 | Rodriguez et al.(2012) | 0.66 | 0.61 - 0.71 | 0.67 | 0.61 - 0.71 |
| 8 | Vasudev et al.(2011) | 0.65 | 0.60 - 0.70 | 0.65 | 0.60 - 0.70 |
| 9 | Le et al.(2008) | 0.68 | 0.63 - 0.72 | 0.69 | 0.64 - 0.74 |
| 10 | Tate et al.(2005) | 0.65 | 0.61 - 0.70 | 0.65 | 0.60 - 0.70 |
| OS | | | | | |
| No. | Model | iAUC | 95% CI | Harrell's C | 95% CI |
| 1 | Zeng et al.(2016) | 0.67 | 0.61 - 0.73 | 0.73 | 0.66 - 0.79 |
| 2 | Pelissier et al.(2016) | 0.69 | 0.62 - 0.76 | 0.75 | 0.68 - 0.81 |
| 3 | Morimoto et al.(2016) | 0.69 | 0.63 - 0.75 | 0.74 | 0.67 - 0.79 |
| 4 | Mahdi et al.(2015) | 0.65 | 0.59 - 0.71 | 0.70 | 0.63 - 0.76 |
| 5 | Pelissier et al.(2014) | 0.67 | 0.61 - 0.74 | 0.74 | 0.67 - 0.80 |
| 6 | Furukawa et al.(2013) | 0.68 | 0.61 - 0.74 | 0.72 | 0.66 - 0.79 |
| 7 | Rodriguez et al.(2012) | 0.68 | 0.62 - 0.74 | 0.74 | 0.67 - 0.80 |
| 8 | Vasudev et al.(2011) | 0.70 | 0.64 - 0.76 | 0.73 | 0.66 - 0.80 |
| 9 | Le et al.(2008) | 0.70 | 0.64 - 0.76 | 0.76 | 0.70 - 0.82 |
| 10 | Tate et al.(2005) | 0.69 | 0.63 - 0.75 | 0.72 | 0.65 - 0.79 |
